# Reduced GluN1 in mouse dentate gyrus is associated with CA3 hyperactivity and psychosis-like behaviors

**DOI:** 10.1038/s41380-018-0124-3

**Published:** 2018-07-23

**Authors:** Amir Segev, Masaya Yanagi, Daniel Scott, Sarah A. Southcott, Jacob M. Lister, Chunfeng Tan, Wei Li, Shari G. Birnbaum, Saïd Kourrich, Carol A. Tamminga

**Affiliations:** 1 Department of Psychiatry, University of Texas Southwestern Medical School, Dallas, TX 75390 USA; 2 Yale University, School of Medicine, 333 Cedar Street, New Haven, CT 06510 USA; 3 Present Address: Department of Neuropsychiatry, Kindai University Faculty of Medicine, Osaka, Japan; 4 Present Address: Yale University, School of Medicine, New Haven, CT USA

**Keywords:** Diseases, Neuroscience

## Abstract

Recent findings from in vivo-imaging and human post-mortem tissue studies in schizophrenic psychosis (SzP), have demonstrated functional and molecular changes in hippocampal subfields that can be associated with hippocampal hyperexcitability. In this study, we used a subfield-specific GluN1 knockout mouse with a disease-like molecular perturbation expressed only in hippocampal dentate gyrus (DG) and assessed its association with hippocampal physiology and psychosis-like behaviors. First, we used whole-cell patch-clamp recordings to measure the physiological changes in hippocampal subfields and cFos immunohistochemistry to examine cellular excitability. DG-GluN1 KO mice show CA3 cellular hyperactivity, detected using two approaches: (1) increased excitatory glutamate transmission at mossy fibers (MF)-CA3 synapses, and (2) an increased number of cFos-activated pyramidal neurons in CA3, an outcome that appears to project downstream to CA1 and basolateral amygdala (BLA). Furthermore, we examined psychosis-like behaviors and pathological memory processing; these show an increase in fear conditioning (FC), a reduction in prepulse inhibition (PPI) in the KO animal, along with a deterioration in memory accuracy with Morris Water Maze (MWM) and reduced social memory (SM). Moreover, with DREADD vectors, we demonstrate a remarkably similar behavioral profile when we induce CA3 hyperactivity. These hippocampal subfield changes could provide the basis for the observed increase in human hippocampal activity in SzP, based on the shared DG-specific GluN1 reduction. With further characterization, these animal model systems may serve as targets to test psychosis mechanisms related to hippocampus and assess potential hippocampus-directed treatments.

## Introduction

Schizophrenia psychosis (SzP) is a chronic disabling brain disorder defined by its psychotic features and rich clinical phenomenology [1], with emerging biological clues pointing up potential brain mechanisms [2–9]. Establishing the neural basis of disease in disorders like schizophrenia is necessary for authenticating disease definition and discovering molecular targets for successful treatment [10, 11]. Neural alterations have been identified across many brain regions in SzP, with findings that appear to associate dysfunction of the prefrontal cortex with cognitive impairments [6]; and hyperactivity in hippocampus [2–5] with psychosis [2, 12]. Interestingly, SzP-imaging studies demonstrate that hyperactivity in the hippocampus co-occurs with widespread neocortical hypoactivity [13]. The hippocampal subfields that appear most involved with molecular and cellular changes in schizophrenia are the dentate gyrus (DG) and the cornu ammonis 3 (CA3) [7]; while the cornu ammonis 1 (CA1) appears to be associated most strongly with the expressed in vivo hyperactivity [3, 4]. In SzP, the DG exhibits several molecular alterations that indicate reduced efferent excitatory signaling to CA3, including decreased neurogenesis [14] and reduced GluN1 expression [15–18]. Most recently in SzP, we reported reduced GluN1 protein selectively in DG [22]. Since the DG plays a crucial role in pattern separation, these molecular changes may be the biological substrates for impaired pattern separation performance already documented in humans with SzP [23]. These new findings combined with reported alterations in hippocampus-mediated behavior [24], function [25, 26], tissue pathology [22, 28], and anatomy [29, 30] are supportive of and consistent with the mounting interest in the hippocampus as a target for SzP pathology.

Because of the distinctive unidirectional neural transmission within the trisynaptic pathway [31, 32], activity changes in a proximal hippocampal subfield like DG would be expected to impact activity-dependent processes in downstream subfields, and especially in CA3. We have reported changes in activity-dependent molecular markers in human post-mortem schizophrenia CA3 tissue (increased GluN2B-containing NMDA receptors and increased PSD95), some of which could represent adaptive changes to decreased afferent stimulation from the mossy fiber pathway [7]. These molecular changes implicate increased synaptic remodeling associated with synaptic strengthening, and are consistent with the discovery of increased dendritic spines on CA3 pyramidal neuronal apical dendrites in SzP [7]. These cellular and molecular changes in CA3 could underlie the hippocampal hyperactivity detected in in vivo imaging studies in schizophrenia, especially if transmitted downstream to CA1 [2–5]. This SzP disease model system suggests that elevated neuronal activity in CA3 could cause mistakes of association and the generation of memories with psychotic constructs, defects that are transmitted to CA1 and consolidated within neocortical regions [33, 34].

Using an animal model system that recapitulates human tissue schizophrenia findings could provide a pivotal resource for testing the functional outcomes of the tissue pathology and establishing causal relationships between identified tissue pathology and psychosis-related behaviors. Using a DG-specific GluN1 KO mouse as a disease-relevant model system [35], we tested cellular activity in DG and CA3, and analyzed specific animal behaviors relevant to psychosis. We hypothesized that reduced DG GluN1 protein in this knockout (KO) would generate DG hypoactivity and CA3/CA1 hyperactivity, thereby leading to behavioral changes relevant to psychosis. A previous report has already shown that DG-specific GluN1-KO animals perform poorly on tasks that require pattern separation [35], a known cognitive characteristic in individuals with schizophrenia [23]. We examined this genetically manipulated mouse by first demonstrating the presence of increased CA3 pyramidal cell activity associated with the DG-specific GluN1 depletion and then by demonstrating psychosis-like behaviors in the mice. To confirm causality, we show that an excitatory designer receptor exclusively activated by designer drugs (DREADD) vector placed in CA3 pyramidal neurons generates similar psychosis-like behaviors in the mouse.

## Methods

### Animal preparations

We generated DG-GluN1 KO mice by crossing POMC-Cre mice with floxed-GluN1 mice, as previously established [35]. DG-GluN1 KO mice and littermate controls (cont) 11–24 weeks old were used for behavioral studies and electrophysiology. DREADD studies utilized 6–8-week-old male C57BL/6J mice, purchased from the UTSW Wakeland Breeding facility; animals underwent behavioral testing at 8–10 weeks of age. All experiments followed institutional guidelines, approved by The Institutional Animal Care and Use Committee at UT Southwestern.

### Electrophysiology

Transverse hippocampal slices (350 μm) from control (cont) and DG-GluN1 KO mice were cut tangentially to the longitudinal axis of the hippocampus. Slices were recovered in a holding chamber for at least 1 h before use. During slicing (0–2 °C) and recordings (at 24.5–25.5 °C), slices were superfused with artifical cerebro-spinal fluid (ACSF) saturated with 95% O_2_/5% CO_2_ and containing (in mM): 119 NaCl, 2.5 KCl, 1.0 NaH_2_PO_4_, 4 MgSO_4_, 4 CaCl_2_, 26.2 NaHCO_3_, and 11 glucose. Pyramidal cells in the CA3 field were visualized using infrared-differential interference contrast optics. Synaptically evoked excitatory post-synaptic currents (EPSCs) were measured using a Multiclamp 700B amplifier (Molecular Devices, Foster City, CA). Spontaneous EPSCs (>250 per cell) were collected and analyzed using Minianalysis software (Synaptosoft, Decatur, GA) and verified visually before calculating frequency and amplitude parameters.

Recording electrodes (3–5 MΩ) contained (in mM): 120 Cs-gluconate, 20 KCl, 10 HEPES, 0.2 EGTA, 2 MgCl_2_, 4 MgATP, and 0.3 NaGTP. Afferents were stimulated at 0.05 Hz by a glass monopolar microelectrode filled with ACSF that was always positioned in the granular cell layer of the DG or in the DG hilus. Data were filtered at 2 kHz, digitized at 10 kHz, and collected and analyzed using Clampex 10.3 software (Clampex 10.3.0.2, Molecular Devices). Membrane potentials of CA3 neurons ranged between −75 and −65 mV. Series resistances ranged from 10 to 20 MΩ and input resistances (Ri) were monitored on-line with a 40 pA/150 ms current injection given before every stimulus. Only cells with a stable Rs (Δ < 15%) for the duration of the recording were kept for analysis. For further details, see supplemental methods.

### Immunohistochemistry

Usual immunohistochemical methods detected cFos and the placement of the DREADDS. Detailed methods, in the supplemental materials.

### Animal surgery/DREADDs

Usual surgical methodology was used to place the DREADD vector; details are included in the supplemental materials. Clozapine-N-oxide (CNO) was given once 30 min prior to testing; for tests performed over a period of several days, CNO was given 30 min prior to testing on each test day.

### Molecular analyses

Western blotting was performed to quantify protein levels. Methodological details are found in the supplemental materials.

### Animal behaviors

Mice were maintained with ad libitum food and water on a 12/12 light–dark cycle. All behaviors were conducted during the light phase. Each group contained 8–20 animals. All behavior raters were blind to the mouse genotypes. Additional methodological details are fiund in the supplemental materials.

#### Prepulse inhibition

Startle was measured using a San Diego Instruments SR-Lab Startle Response System (San Diego, CA). Testing consisted of 40-startle stimuli (120 dB) preceded (100 ms) by a prepulse stimulus (20 ms). Prepulse intensities were 0, 2, 4, 8, or 12 dB above the background noise (70 dB) and presented within a pseudorandom order with an average inter-stimulus interval of 15 s (range 7–23 s).

#### Passive avoidance behavior

The mice were placed in a brightly lit side of a shuttle box (Med Associates, Inc., St. Albans, VT). When the door opened to the dark side and the animal entered the dark compartment, they received two 1 s, 0.5 mA footshocks. Twenty-four hours later, the procedure was repeated without footshocks. The latency to enter the dark compartment was measured on both days.

#### Fear conditioning

Fear conditioning (FC) was measured in automated boxes (Med Associates, St. Albans, VT). For testing with the DG-GluN1 KO mice, mice received five cue presentations (10 s white noise, 80 dB, 30 s inter-trial interval), which co-terminated with a footshock (1 s, 0.5 mA). For DREADD studies, mice were presented with three cue-shock pairings. Contextual fear was measured in the same chamber 24 h later but without footshock. Cued fear conditioning was measured 48 h after training, in a modified chamber (plastic floor, “V-Ceiling,” vanilla scent). Tones were presented without shock and freezing was measured during the tone. Freezing behavior was scored automatically using Med Associates software [36, 37].

#### Morris Water Maze test

Mice were trained to find a fixed submerged platform in a pool of opaque water (144 cm, diameter) with four training trials per day (1 min swimming time, inter-trial interval of 30–45 min) for 13 days. On the probe day, occupancy time (%) in the target quadrant was compared to all other quadrants and the platform crossings in the target quadrant were compared to a similar area in all other quadrants.

#### Locomotor activity

Individual mice were placed into clean home cages with a small amount of bedding. Locomotor activity was collected in 5-min bins in the dark (San Diego Instruments, San Diego, CA, USA) for 120 min.

#### Social memory

Mice were placed into an empty cage for adaptation, after which a 4-week-old male C57BL/6J mouse was placed in the cage for 2 min; the time the resident mouse spent in contact/sniffing, following, nosing/grooming, or pawing/general inspection was measured. The procedure was repeated in 24 h, introducing the same juvenile mouse to the same resident. The decrement in engagement time was taken to represent social memory. Social memory was tested in both the KO and the DREADD animal model systems.

### Statistical analysis

All statistical analyses used GraphPad Prism software (San Diego, CA, USA). Significance was set at *p* < 0.05. Outcomes for locomotion were tested using two-way analysis of variance (ANOVA) (genotype × time) and an unpaired *t*-test. Two-way ANOVAs were also used to assess differences in prepulse inhibition (PPI) (decibel × genotype/group), FC (situation × genotype/group), cFos-positive nuclei along the sequential coronal sections (Bregma coordinates × genotype), AMPA and NMDA receptor EPSCs (stimulus intensity × amplitude), and paired-pulse ratio (PPR × inter-stimulus interval). Either an uncorrected Fisher least significant difference, Bonferroni’s multiple comparison, or Sidak’s multiple comparisons post hoc test were performed when significance was found with ANOVAs. An upaired *t*-test was used to test group differences (passive avoidance (PA), MWM, social memory, western blots, total cFos, and the 30 ms time point of the PPR). One-way ANOVAs were also used for electrophysiological analysis. Data are presented as mean ± SEM in Table 1 (supplement). Full statistical outcomes are specified in the figure legends. Error bars in figures represent SEM.

## Results

### Dentate gyrus characteristics in the GluN1 KO mouse

#### Molecular measures: dentate gyrus

Protein quantification showed a decrease in GluN1 protein in the KO mouse, confined to the DG compared with its littermate control (cont) (*t* = 7.54, df_1,12_, *p* < 0.0001) and not present in CA3 (*t* = 0.84, df_1,12_, *p* = 0.42) or CA1 (*t* = 0.21, df_1,12_, *p* = 0.84), as previously reported [35]. As well, the KO mouse tissue showed decreased GluN2A (*t* = 6.99, df_1,12_, *p* < 0.0001) and GluN2B (*t* = 8.65, df_1,12_, *p* < 0.0001) subunits limited to DG. (Suppl. Table). POMC-Cre immunohistochemistry (IHC) showed that the POMC construct is limited to DG and hypothalamus in the KO animal (data not shown).

#### Electrophysiological measures: dentate gyrus

**Fig. 1:**
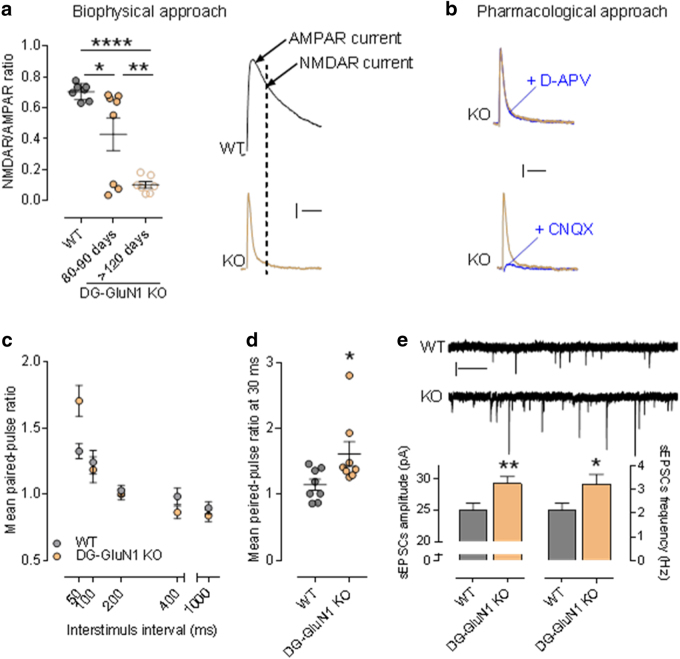
**(a)** Left panel, NMDAR/AMPAR ratio in cont and DG-GluN1 KO. AMPAR- and NMDAR-EPSC amplitudes are extracted from the dual component obtained at +40 mV, at 10 and 50 ms post-stimulus, respectively. Biophysical analysis of the dual component at +40 mV showed that NMDAR-mediated current in DG granular cells (dash line on the right panel) is absent in DG-GluN1 KO (4–5 months old). Note that the NAR decrease observed at 80–90 days is driven by 3/8 cells that were not exhibiting any NMDAR-mediated current (measured at 50 ms, dash line). Hash marks on left panel indicate group means ± SEM. (One-way ANOVA: *F* (2, 19) = 18.41: ****p < 0.0001; post hoc test: *p < 0.05; **p < 0.01). Calibration: 50 ms, 20 pA. (**b)** D-APV at 50 μM did not have any effect on evoked EPSC in DG-GluN1 KO (dual component obtained at 40 mV), indicating that NMDAR-mediated current is not present. AMPAR blockade with CNQX 10 μM almost totally eliminated evoked EPSC. Calibration: 50 ms, 20 pA. (**c)** Mean paired-pulse ratio values in CA3 pyramidal neurons from DG-GluN1 KO mice (*n* = 15 cells, 4 mice) is increased at an inter-stimulus interval of 50 ms compared to cont (*n* = 17 cells, 4 mice) (two-way ANOVA, Interaction PPR × inter-stimulus interval: *F*(4, 120) = 6.328, *p* = 0.0001; post hoc test at 50 ms: ***p < 0.01). (**d)** Mean paired-pulse ratio values in CA3 pyramidal neurons from DG-GluN1 KO mice (*n* = 8 cells, 3 mice) is also increased at 30 ms inter-stimulus intervals compared with neurons from cont (*n* = 8 cells, 3 mice) (*t*(14) = 2.294, *p < 0.05). (**e)** Top panel, sample traces of sEPSCs from neurons in cont and DG-GluN1 KO (KO) groups. Calibration: 1 s, 20 pA. Bottom panel, spontaneous EPSCs amplitude and frequency are increased in DG-GluN1 mice (*n* = 23 cells, 5 mice) compared with cont (*n* = 21 cells, 6 mice). Amplitude: *t*(42) = 2.819, **p = 0.007; frequency: *t*(42) = 2.194, *p = 0.034

In the GluN1 DG-KO mice, DG granule cells do not exhibit any NMDAR-mediated current (Fig. 1). Specifically, we measured the NMDAR/AMPAR ratio (NAR) in DG granular cells at two time points during development, 80–90 and >120 days of age. Consistent with the literature [35], both biophysical and pharmacological approaches show that NMDAR-mediated current in granule cells of the DG-GluN1 KO mouse decreases during the first few months of development and is totally eliminated by 4 months of age (Fig. 1a, b). These observations are consistent with the loss of GluN1 protein in the DG granule cell over the same time period. To determine whether this genetic manipulation altered the capability for granule cells to convey information, we measured the responses to paired-pulse stimulation, a standard paradigm to test for changes in glutamate presynaptic release probability (*p*_r_). We show that granule cells from DG-GluN1 KO mice exhibit an enhanced PPR at short inter-stimulus intervals (50 ms) (Fig. 1c), indicating a decrease in *p*_r_. Further investigation of different groups of animals confirmed these findings and showed that PPR is similarly increased at 30 ms inter-stimulus interval (Fig. 1d). Furthermore, both amplitude and frequency of spontaneous activity-dependent release of glutamate at the MF-CA3 synapses (sEPSCs) are enhanced in GluN1 DG-KO mice (Fig. 1e).

### CA3 hippocampal characteristics in the GluN1 KO mouse

#### Electrophysiological measures: CA3

**Fig. 2:**
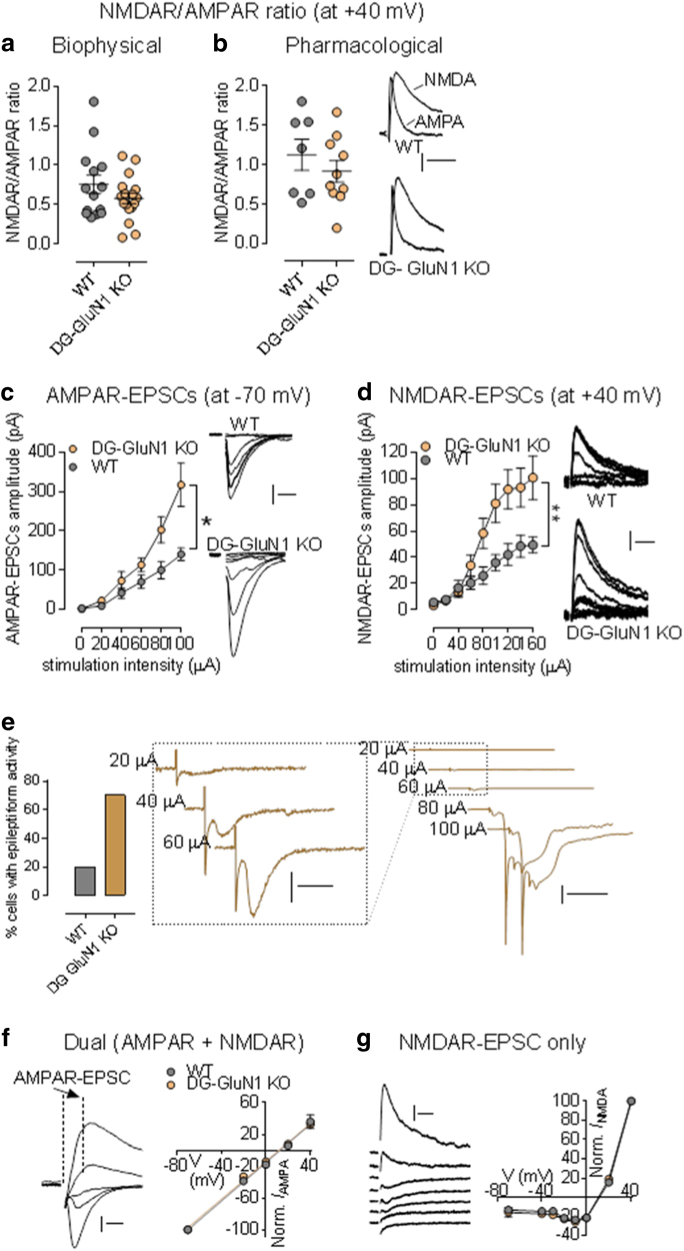
**(a**, **b)** NMDAR/AMPAR ratio values from neurons in cont (gray circles; *n* = 8 cells, 5 mice) and DG-GluN1 KO mice (orange circles; *n* = 7 cells, 6 mice). Hash marks indicate mean values ratio in cont and DG-GluN1 KO. Both biophysical (**a**) and pharmacological (**b**) approaches showed that NMDAR/AMPAR ratio in cont is similar to that of found in DG-GluN1 KO mice (*p* > 0.05). For biophysical approach, AMPAR- and NMDAR-EPSC amplitudes are extracted from the dual component obtained at +40 mV, at 10 and 50 ms post-stimulus, respectively. For pharmacological approach: D-APV at 50 μM was used to extract AMPAR-mediated current. (**b**) Right panel: sample EPSCs from cont (WT) and DG-GluN1 KO mice. Calibration: 50 ms, 20 pA. (**c**, **d)** Both AMPAR- (**c**) and NMDAR-mediated transmission (**d**) at MF-CA3 synapses are increased. AMPAR-mediated transmission was assessed at −70 mV, and NMDAR-mediated current was elicited at +40 mV. Right panels for (**c)** and (**d)**: example of AMPAR- (calibration: 10 ms, 50 pA) and NMDAR-EPSCs traces (calibration: 50 ms, 20 pA) from a cont and a DG-GluN1 KO neuron over the stimulus range 0, 20, 40, 60, 80, and 100 μA. Cont, *n* = 10 cells, 4 mice; DG-GluN1 KO, *n* = 16 cells, 4 mice. Two-way ANOVA: genotype and genotype × stimulation interaction effects for both AMPAR- (genotype effect: *F*(1, 24) = 4.312, *p = 0.048, and interaction effect: *F*(4, 96) = 3.850, **p = 0.006) and NMDAR-EPSCs (genotype effect: *F*(1, 17) = 5.336, *p = 0.033, and interaction effect: *F*(8,136) = 6.160, ***p < 0.0001). (**e)** Left panel, percentage of cells demonstrating eplileptiform activity indicated by more frequent late burst EPSC recruitment achieved at lower stimulus intensities was significantly higher at DG-GluN1 KO mice (12/16 cells) compared with cont (2/10 cells). Right panel, sample traces from CA3 pyramidal neurons from DG-GluN1 KO mice. Calibration in left: 20 ms, 50 pA; calibration in right: 100 ms, 1 nA. (**f)** Left, examples of evoked dual EPSCs at membrane potentials from −80 to +40 mV. Calibration: 10 ms, 50 pA. Right, *I*–*V* relationship for AMPAR-EPSCs (measured by extracting the AMPAR current from the dual component at 10 ms post stimulus) in cont and DG-GluN1 KO mice (*n* = 8–10 cells, 5 mice in each group). The lines represent the linear regression (*r* = 0.99 for each group). (**g)** Left, examples of evoked NMDAR-mediated EPSCs at membrane potentials from −80 to +40 mV. Calibration: 50 ms, 20 pA. Right, *I*–*V* relationship for NMDAR-mediated EPSCs in cont and DG-GluN1 KO mice (*n* = 5–11 cells, 3–4 mice in each group). Holding potentials were not corrected for liquid junction potential. Data are represented as means ± SEM

Excitatory glutamate transmission in CA3 of DG-selective GluN1 KO mice is enhanced compared to cont mice (Fig. 2). Using whole-cell patch-clamp recordings, we first examined the contribution of excitatory glutamate receptors, AMPA and NMDA receptors, to synaptic transmission at the MF-CA3 pyramidal neuronal synapses. Using both biophysical and pharmacological approaches, we found no change in NAR ratio in DG-GluN1 KO mice when compared to cont mice (Fig. 2a, b). However, when both receptor-mediated currents were assessed separately, we found a significant increase in both AMPAR- and NMDAR-mediated post-synaptic excitatory currents (Fig. 2c, d). These data show that decreased DG granular cell GluN1 protein in DG-GluN1 KO mice, loss of DG NMDAR-mediated current, and decreased *p*_r_ at the MF-CA3 synapses are associated with increased glutamatergic synaptic strength at the MF-CA3 synapses. Moreover, we observed that this increased excitatory glutamatergic transmission in CA3 translates into hyperexcitability of CA3 pyramidal neurons in DG-GluN1 KO mice. In particular, increasing stimulus intensity triggered spikes and recruited late burst EPSCs more routinely and at a lower intensity in DG-GluN1 KO mice (12/16 cells: 75%) compared to wild-type control slices (2/10 cells: 20%, Fig. 2e). Since these observed alterations in synaptic activity can result from functional changes (i.e., altered subunit composition) in CA3 NMDARs and AMPARs, we examined the current–voltage relationships for both receptors. Evoked AMPAR-mediated EPSCs measured from the dual component at 10 ms post stimulus (Fig. 2f) and NMDAR-mediated EPSCs (Fig. 2g) were unchanged in DG-GluN1 mice compared to cont mice, indicating that the hyperexcitability observed in CA3 is not due to changes in AMPARs and NMDARs function.

#### Cellular measures: CA3

**Fig. 3:**
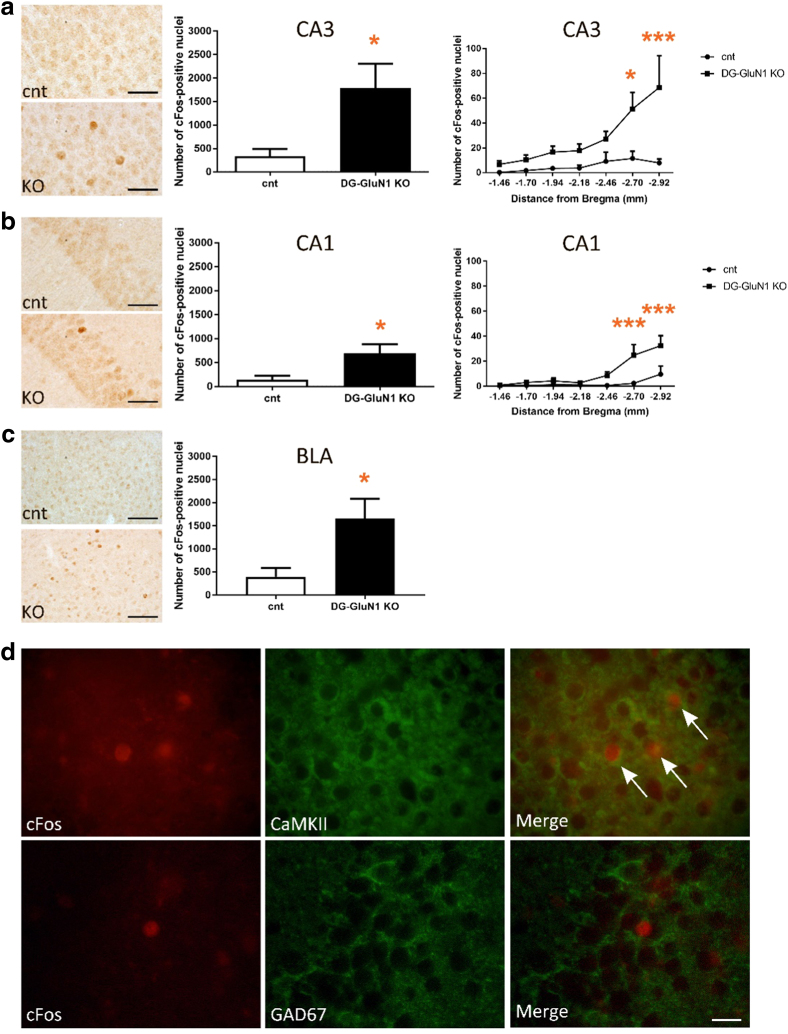
**a** Increased number of cFos-positive nuclei in hippocampal CA3. Left panel, representative images in cont and DG-GluN1 KO brains. Middle panel, the total number of cFos-positive nuclei was significantly increased in CA3 (*t*(8) = 2.665, *p = 0.02). Right panel, the number of cFos-positive nuclei over the rostral (dorsal)-caudal (ventral) axis of CA3 of the hippocampus (−1.46 to −2.92 mm from Bregma). Two-way ANOVA analyses show significant genotype, hippocampal rostral-caudal axis, and genotype × hippocampal rostral-caudal axis interaction in CA3 (genotype effect: *F*(6, 56) = 4.635, ***p = 0.0007; hippocampal rostral-caudal axis effect: *F*(1, 56) = 24.53, ****p < 0.0001; and interaction effect: *F*(6, 56) = 2.634, *p = 0.0254). Post hoc comparison further demonstrates significant increased cFos-positive nuclei in the caudal (ventral) CA3 (*p = 0.01 at −2.70 mm from Bregma, ****p < 0.0001 at −2.92 mm from Bregma). **b** Increased cFos-positive nuclei in hippocampal CA1. Left panel, representative images. Middle panel, increased total number of cFos-positive nuclei in CA1 (*t*(8) = 2.614, *p = 0.03). Right panel, the number of cFos-positive nuclei over the rostral-caudal axis of CA1 of the hippocampus (−1.46 to −2.92 mm from Bregma). Two-way ANOVA analyses show significant genotype, hippocampal rostral-caudal axis, and genotype × hippocampal rostral-caudal axis interaction in CA1 (genotype effect: *F*(6, 56) = 8.286, ****p < 0.0001; hippocampal rostral-caudal axis effect: *F*(1, 56) = 18.34, ****p < 0.0001; and interaction effect: *F*(6, 56) = 3.374, **p = 0.0066). Post hoc comparison further demonstrates significant increased cFos-positive nuclei in the caudal CA1 (***p = 0.0007 at −2.70 mm from Bregma, ***p = 0.0005 at −2.92 mm from Bregma). **c** Increased number of cFos-positive nuclei in basolateral amygdala (BLA, −0.70 to −2.30 mm from Bregma, *t*(8) = 2.709, *p = 0.02). Left panel, representative images. **d** The majority of cFos-positive nuclei in hippocampal pyramidal layer in CA3 subfield were located within CaMKII-positive excitatory neurons, but not within GAD-67-positive inhibitory neurons

To test whether the increase in AMPAR- and NMDAR-mediated post-synaptic currents alters overall cellular activity in CA3, we analyzed cFos regionally in the DG-GluN1 KO mice. We found increased total number of cFos-positive nuclei in the pyramidal layer in ventral hippocampal CA3 along the rostral-caudal axis in the KO compared with the cont mouse (Fig. 3a). Further analyses show a similar pattern of increased number of cFos-positive neurons in CA1 (Fig. 3b), but no detectable changes in granule cells in DG (data not shown). Curiously, increased cFos-containing neurons were also found in BLA, regionally clustered (Fig. 3d) but not in basal ganglia.

Double-staining IHC experiments with excitatory vs inhibitory cell markers confirmed that the majority of cFos-positive nuclei in the CA3 pyramidal layer were localized to the CaMKII-positive excitatory neurons, and were not detected in GAD67-positive inhibitory interneurons (Fig. 3d).

### Behavioral characteristics in the GluN1 KO and CA3 DREADD mouse

We assessed several correlates of attention and cognitive deficits typically observed in humans with psychosis (PPI, MWM, social memory (SM)) and explicit associative learning tasks (FC, PA), first in the DG-selective GluN1 KO.

#### Prepulse inhibition

**Fig. 4:**
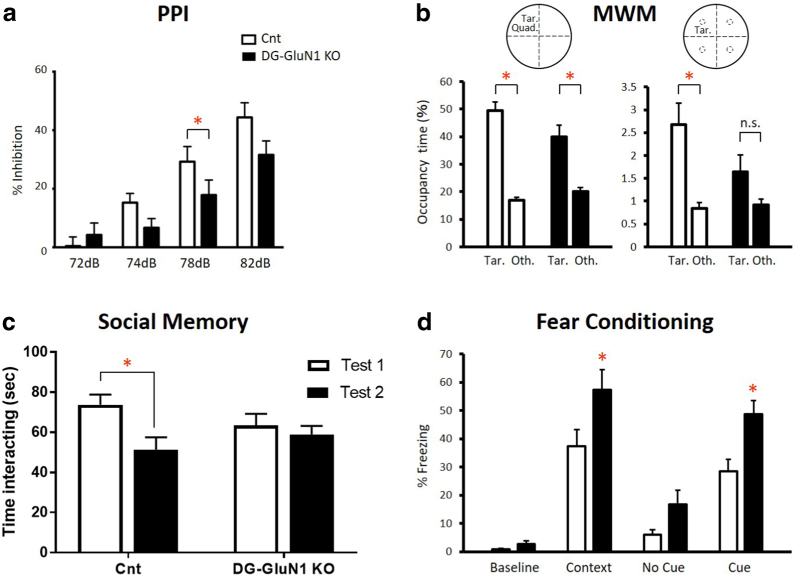
**(a)** Prepulse inhibition (PPI) was reduced in DG-GluN1 KO mice compared with cont littermates. Two-way ANOVA (genotype × dB level): *F*(3, 108) = 3.299, *p = 0.0232. Decible level: *F*(3, 108) = 55.15, *p* < 0.0001; genotype: *F*(3, 108) 2.127, *p* = 0.1534. Post hoc test at 78 dB, *p = 0.039 and a trend for significance at 76 dB, *p* = 0.066. (**b)** Morris Water Maze. Number of crossings of the target quadrant (left) vs the mean of the other non-target quadrants (right) on day 13 probe test. The cont mice (left, white) remembered where the platform was located (target quadrant relative to average of the other quadrants: *t* = 9.715, df_1,38_, *p* < 0.0001 or platform area relative to average of the parallel areas in the other quadrants: *t* = 3.792, df_1,38_, *p* = 0.0005); whereas, DG-GLuN1 KO mice (right, black) failed to remember accurately where the target was located (target quadrant relative to average of the other quadrants: *t* = 4.370, df_1,34_, *p* < 0.0001; platform area relative to average of the corresponding areas in the other quadrants: *t* = 1.831, df_1,34_, *p* = 0.0758). Platform vs other. (**c)** Social memory (SM). In contrast to cont mice (*t* = 2.81, df_1,24_, *p* = 0.02), DG-GluN1 KO mice showed no decrease in interaction time upon re-exposure to a juvenile mouse 24 h following initial exposure (*t* = 0.62, df_1,24_, *p* = 0.79). (**d)** Fear conditioning: The unconditioned DG-GluN1 KO mice did not show elevated freezing in a new context (*t* = 1.37, df_1,28_, *p* = 0.18). However, when analyzing genotype × situation with two-way ANOVA, there was a significant effect of situation (*F*(2,28) = 34.43, *p* < 0.0001) and genotype (*F*(1,14) = 20, *p* = 0.0005) (interaction genotype × situation: *F*(2,28) = 0.4838, *p* = 0.6215). Post hoc tests showed a significant increase in freezing in the same context (*p* = 0.0160) and in the cued context (*p* = 0.0147) without difference in the new context (*p* = 0.1792)

Similar to humans with psychosis, DG-GluN1 KO mice showed reduced PPI in comparison with cont mice (Fig. 4a).

#### Morris Water Maze

On the day 7 probe trial, only the control mice showed a significant preference for the target quadrant vs the mean of the other 3 quadrants. On the day 13 probe trial, both the KO mice and their cont littermates spent significantly more time in the target quadrant (Fig. 4b). Cont mice crossed the platform area significantly more times than parallel areas in other quadrants on the day 13 probe test; while the KO mice did not show a significant increase in crossing over the target platform in either probe test (Fig. 4b). However, while cont mice also spent significantly more time in the platform area than in the parallel areas in the other quadrants on the second probe test, the KO mice did not (Fig. 4b), suggesting a deficit in spatial memory.

#### Passive avoidance

In the acquisition trial of the PA test, there was no difference between the KO and cont mice in the latency to enter the dark compartment (*t* = 0.15, df_1,37_, *p* = 0.44). But on the test day, the KO mice showed increased latency to enter the dark compartment where they had previously been shocked (*t* = 2.63, df_1,37_, *p* = 0.01) (data not shown).

#### Fear conditioning

Before training, there was no difference in percent freezing between the KO mice and cont littermates. Twenty-four hours after training, the KO mice display increased freezing in the training context (contextual fear) and to the tone compared to cont (Fig. 4d).

#### Social memory

The DG-selective GluN1 KO mice and cont littermates displayed similar levels of social engagement when introduced to a juvenile intruder mouse. However, upon re-exposure 24 h later, cont but not KO littermates exhibit a decrease in social interaction time, which indicates impaired social recognition in KO mice (Fig. 4c).

#### Locomotor activity

No difference in locomotor activity was observed between the DG GluN1 KO mice and cont over 120 min (*t* = 0.64, df_1,36_, *p* = 0.53) (data not shown).

#### Behavioral outcomes with CA3 DREADD

**Fig. 5:**
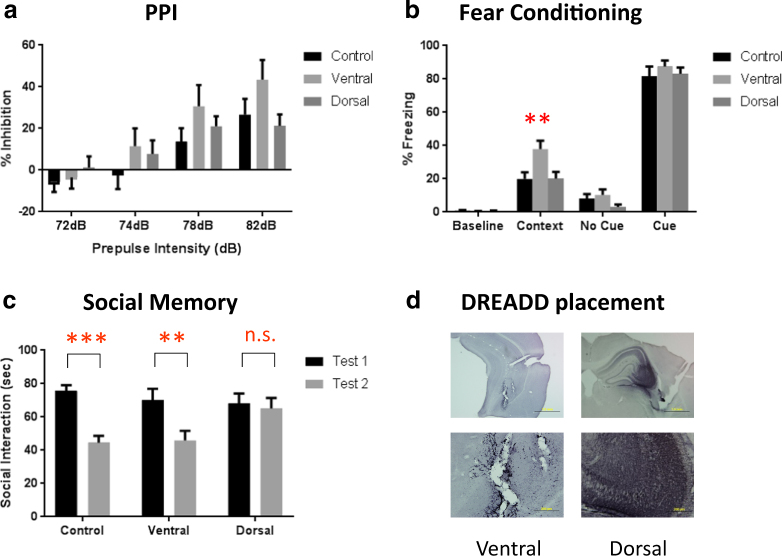
Behavioral analysis after DREADD-induced excitation of CA3. (**a)** Prepulse inhibition. Two-way ANOVA revealed a main effect of prepulse intensity (*F*(3, 81) = 42.08, *p* < 0.0001) and a prepulse × group interaction (*F*(6, 81) = 2.224, *p* = 0.049). However, post hoc analysis shows no difference between any group at any specific prepulse intensity. (**b)** Fear conditioning. There is a significant main effect of region (*F*(2, 103) = 4.887, *p* = 0.0094), and post hoc analysis revealed a significant increase in contextual fear conditioning after activation of the ventral CA3 (*p* = 0.0016). Activation of this region did not affect baseline freezing or cued fear, and activation of the dorsal CA3 did not alter any fear-related behavioral measure. (**c)** Social memory. Normal social recognition is demonstrated by a significant decrease in interaction time between the first and second tests and was present in control animals (*t* = 4.237, df_1,54_, *p* < 0.001). Ventral CA3-activated DREADD mice significantly decreased interaction time (*t* = 2.994, df_1,54_, *p* < 0.01), whereas activation of the dorsal CA3 with the excitatory DREADD impaired this recognition, resulting in no decrease in interaction time between the two tests (*t* = 0.3649, df_1,54_, *p* = 0.717). (**d)** Verification of DREADD placement based on mCherry staining. DAB signaling was enhanced by the addition of nickel sulfate to give the mCherry staining a black color, distinguishing the specific mCherry signal from background gliosis present as a result of the AAV infusion. Images represent the ventral (left) and dorsal (right) CA3 at 4× (top) and 20× (bottom). **, *** represents *p* < 0.01, 0.001, respectively

Here, we set out to establish causality between CA3 hyperactivity and behavioral outcomes. We expressed a Gq-coupled DREADD vector virally in pyramidal neurons in dorsal or ventral CA3 hippocampus and examined PPI, FC, and social cognition. We found no significant change in PPI with a single CNO administration (Fig. 5a). However, DREADD activation of ventral (not dorsal) hippocampus increased FC (Fig. 5b); whereas that same activation of dorsal hippocampus (not ventral) impaired social memory (Fig. 5c). These behavioral outcomes demonstrate the behavioral phenotype associated with CA3 hyperactivity in both FC and social memory; and show that modulation of psychosis-like behaviors will vary regionally within CA3 activation. Anatomic placement of the DREADD constructs were verified in all animals used for data analysis (Fig. 5d).

## Discussion

The literature contains several different SzP model systems based on GluN1 KO mice, one where the GluN1 KO is brain-wide [7, 38] and another where the KO is selective for inhibitory interneurons [39, 40]. Here, we examined the characteristics of a DG-selective GluN1 KO mouse as a model for maladaptive neuroadaptations, based on back translation from disease pathology derived from human SzP brain tissue. Whole-cell recording of granule cells, an approach that provides high-neuronal specificity, shows that NMDAR-mediated current is entirely eliminated in recorded DG cells when the DG-GluN1 KO mouse is fully developed. Because NMDA receptors function as heteromeric receptors with an essential GluN1 subunit [41, 42], it is likely that the depletion of GluN1 attenuates NMDA receptor formation (supported by the reduction in DG of GluN2A and GluN2B in the KO) and, therefore, is associated with a decreased efferent excitatory activity from DG, consistent with our findings in this study. This genetic manipulation is associated with enhanced NMDA- and AMPA-mediated currents in CA3. The enhanced excitatory post-synaptic excitability at the MF-CA3 synapses translates into elevated pyramidal cell activity in CA3 as shown by enhanced cFos-positive pyramidal cell nuclei in the KO mouse CA3 and its downstream target, CA1. Curiously, we observed related clusters of cFos-activated neurons not only in CA3, CA1 but also in BLA showing downstream hyperactivity in the KO mice, suggesting a forward pathway which could mediate extended effects of hippocampal hyperactivity. These characteristics parallel and extend the findings reported from human SzP tissue, suggesting the plausibility of reduced DG granule cell activity generating increased CA3 pyramidal cell activity, leading to overall hippocampal hyperactivity. With these data, the speculative link between hippocampal hyperactivity and psychosis [2–5] becomes a more plausible scenario, where sustained hippocampal hyperactivity causes mistakes of association and a vulnerability for generating memories with psychotic content, accounting in part for positive symptoms in schizophrenia [33]. However, because DG granular cells in DG-GluN1 KO mouse exhibit a decreased *p*_r_, we speculate that decreased granular cells activity triggers a network-wide homeostatic adaptation, albeit pathological, that aims to re-normalize transmission in the MF-CA3 excitatory transmission (reviewed in ref. [33]). While many necessary experiments remain to test this model, these data do positively align the cellular physiology of hippocampal circuitry with schizophrenia-related pathology.

The behavioral characteristics observed here in the DG-GluN1 KO mouse focus the KO behavioral profile onto psychosis and CA3 function. The impaired PPI and MWM performance along with a deficit in SM are characteristics of mouse models of psychosis reflecting cognitive impairments also observed in SzP [29]. In addition, the animals show an increase in FC and PA behavior, with locomotor activity intact, reflecting CA3 neuronal hyperactivity. The CA3 DREADD mouse also shows a deficit in SM as well as increased FC, which are differentially affected by activation of the dorsal and ventral CA3. The increase in fear learning suggests that where learning is driven by fear or anxiety, as we find here, a persistent psychotic memory could occur. The early findings from these animal model systems are consistent with the general model of psychosis as a learning and memory disorder with deficient DG function and increased CA3 associative function [33, 43]. The data so far support the further testing of the DG-GluN1 KO and hippocampal DREADD vectors as animal systems informative about psychosis pathophysiology.

These outcomes establish the association of reduced excitatory signaling in the DG and increased pyramidal cell activity in CA3 and CA1, and in BLA. These may explain the overall hippocampal and CA1 hyperactivity in SzP, pathophysiology that has gained support from studies carried out in SzP tissue and in vivo imaging [44]. Future experiments will test whether reduced DG activity is causally related to CA3 hyperactivity using an inhibitory DREADD in DG. Moreover, in order to demonstrate relevance to human psychosis, we will assess the action of known antipsychotic drugs on these molecular, cellular, and electrophysiological outcomes. If further studies continue to supports its relevance, the availability of an animal preparation reflective of the human hippocampal dysfunction in SzP [45, 46] will be a significant advantage for studying psychosis in schizophrenia.

## Electronic supplementary material

Supplement
