# Supplementary material for: Reduced GluN1 in mouse dentate gyrus is associated with CA3 hyperactivity and psychosis-like behaviors
Source: Mol Psychiatry. 2018 Jul 23;25(11):2832–43. doi: 10.1038/s41380-018-0124-3 (PMC6344327; doi:10.1038/s41380-018-0124-3)
Supplement: Supplementary file 1 — Supplement [file 41380_2018_124_MOESM1_ESM.docx]

SUPPLEMENTAL TABLE

**Table1. Mean ± SEM and p-values for immunoquantification**

|  | Littermates (mean ± sem) | DG-GluN1 KO (mean ± sem) | p-value |
| --- | --- | --- | --- |
| DG | 2.34 ± 0.07 | 1.66 ± 0.06 | <0.0001 |
| GluN1 CA3 | 1.05 ± 0.08 | 0.95 ± 0.08 | 0.42 |
| CA1 | 2.04 ± 0.27 | 1.97 ± 0.2 | 0.84 |
| DG | 2.51 ± 0.09 | 1.45 ± 0.11 | <0.0001 |
| GluN2A CA3 | 1.02 ± 0.05 | 0.98 ± 0.03 | 0.55 |
| CA1 | 2.09 ± 0.30 | 1.91 ± 0.31 | 0.69 |
| DG | 2.59 ± 0.07 | 1.41 ± 0.12 | <0.0001 |
| GluN2B CA3 | 1.03 ± 0.02 | 0.97 ± 0.03 | 0.18 |
| CA1 | 2.08 ± 0.34 | 1.92 ± 0.34 | 0.75 |

# SUPPLEMENTAL METHODS

**Animal Preparations and Surgical techniques:**

The POMC-Cre mice were originally developed by Dr. Joel Elmquist and Dr. [Lowell BB](http://www.ncbi.nlm.nih.gov/pubmed/?term=Lowell%20BB%5BAuthor%5D&cauthor=true&cauthor_uid=15207242) were kindly given to us by Dr. Jeffrey Zigman (UT Southwestern Medical Center). The floxed-GluN1 mice developed by Dr. Tonegawa were purchased from the Jackson Laboratory (http://jaxmice.jax.org /strain/005246.html). The genotyping of Cre detection was performed as in McHugh TJ (34), and the protocol from the Jackson Laboratory was used for genotyping floxed-GluN1. Mice were housed at UTSW in a vivarium accredited by the Association for Assessment and Accreditation of Laboratory Animal Care in temperature-controlled rooms on a constant 12 h light/dark cycle with *ad libitum* food and water. Experiments were conducted during the light cycle at approximately the same time each day.

For placement of the DREADD vector, after being allowed one week to habituate to the animal housing facility, mice were anesthetized with isoflurance and placed into a stereotaxic apparatus (David Kopf Instruments, Tujunga, CA). Holes were drilled bilaterally into the skull, and infusions of an excitatory DREADD (pAAV-CaMKIIa-hM3D(Gq)-mCherry was a gift from Bryan Roth (Addgene plasmid # 50476)) were given at a volume of 0.15μl per infusion via a Hamilton syringe into the dorsal or ventral CA3. Following each infusion, the syringe was left in place for 5 minutes. Three infusions were given per hemisphere in both the dorsal and ventral CA3. Coordinates relative to bregma used for targeting the dorsal CA3 were: AP -1.7mm, ML ±2.0mm, DV -1.9mm; AP -1.8mm, ML ±2.3mm, DV -2.0mm; AP -2.3mm, ML ±2.5mm, DV -2.5mm. For ventral CA3, coordinates were: AP -2.8mm, ML ±2.9mm, DV -3.0mm; AP -2.9mm, ML ±3.2mm, DV -3.5mm; AP -3.1mm, ML ±3.2mm, DV -3.6mm. Mice were allowed at least six days recovery after surgery before behavioral testing. Clozopine-N-oxide (CNO; 1 mg/kg) or saline was injected i.p. at a volume of 2 ml/kg 30 minutes prior to behavioral testing.

**Electrophysiology:**

Electrophysiology: High Ca2+ and Mg2+ concentrations (4 mM) for extracellular solution was used to reduce cellular excitability and thus to inhibit the epileptiform activity to which the CA3 region is especially prone (as performed in (47-49)). It also allows to better isolate the MF-CA3 EPSC from recurrent associational/commissural (i.e., polysynaptic) EPSCs. For most of the experiments, LY-354740 1μM, a highly selective and potent agonist of group II mGlu (metabotropic glutamate) receptors, was also applied at the end of the experiments to verify that evoked EPSCs were mediated by glutamate release from MF. Kynurenic acid (2 mM) was used to block glutamatergic transmission during slicing, and thereby limit excitotoxic processes. During recordings, ACSF containing picrotoxin (100 μM) was used to block GABAA receptor-mediated inhibitory postsynaptic potentials and CNQX (10 μM) to block AMPARs when recording NMDAR-mediated transmission.

**Animal Behaviors:**

PPI: Mice were placed into the Plexiglass holders and allowed to acclimate to the chamber and background white noise (70 dB) for 5 min. After the acclimation period, seven startle stimuli (120 dB, 40 ms, white noise) were presented.

PA: The experimental apparatus consisted of a brightly lit compartment connected to an identical dark compartment through a motorized sliding door. In the acquisition trial, the animals were placed in the illuminated compartment and were allowed to freely explore for 90 s. After this, the motorized door was lifted and the animals were allowed to cross into the dark chamber. If a mouse did not enter the dark compartment for 1200 s, it was removed from the box and assigned a ceiling score of 1200 s.

FC: Med Associates software (threshold of 20 arbitrary units and minimum freeze duration of 0.5 sec automatically scored freezing behavior.

MWM: In all trials mice were released from pseudo-random assigned start locations, and were allowed to swim until they stopped for 2 s on the platform or 60 sec elapsed. They were manually guided to the platform in the case of failures. To assess spatial learning ability, distinct distal cues were placed on the surrounding walls. On days 7 and 13, a probe test was conducted in the morning with the platform removed. The mice were allowed to swim for 60 sec. At the probe test, mice were assessed for their ability to recognize the radial-quadrant where the platform was located during training and the platform area where the platform was located during training. The swim path and time in each quadrant were recorded (Ethovision, Noldus Information Technology, Leesburg,VA).

**Immunoblotting:**

The frozen samples were homogenized using sonication in RIPA Lysis buffer with proteinase inhibitors (Santa Cruz, CA). Protein concentrations in the subfield homogenates were determined using the BCA assay kit (Pierce, Thermoscientific, IL). Thirty micrograms of protein per sample was resolved on a 7.5% SDS-PAGE gel, transferred to a nitrocellulose membrane, blocked in 5% milk in TBST (50 mM Trisbuffered saline, pH 7.4 and 0.1% Tween-20) and then incubated in primary antibody: GluN1 (1:1000), GluN2A or GluN2B (1:1000).

cFOS: To detect cFos immunohistochemistry, fresh perfused (4% PFA) mouse brain was collected, cryoprotected in 30% sucrose in 0.1M PBS, and sectioned (40 µm; Leica SM2000R). Every 9^th^ 40-µm coronal section of hippocampus was mounted, dried at room temperature, subjected to microwave antigen retrieval (citra solution, BioGenex; 95°C for 10 min), quenched free of endogenous peroxidases in 0.3% H_2_O_2_, and blocked in 3% normal donkey serum, prior to overnight incubation of rabbit anti-cFos polyclonal antibody (1:8000; Thermo Scientific). Primary antibody was detected by sequential incubation with biotinylated donkey anti-rabbit IgG (1:1000, Jackson ImmunoResearch) and avidin-biotin complex (Vector Laboratories). Diaminobenzidine chromogen was used to detect the immunoperoxidase signal. cFos-positive cell nuclei were counted blindly in hippocampal subregions bilaterally, from rostral (dorsal)-to-caudal (ventral) (-1.46 mm to -2.92 mm from bregma), in basolateral amygdala (BLA, -0.70 to -2.30 mm from Bregma) and medial prefrontal cortex (mPFC, 1.54-1.34 from Bregma). For double staining, primary antibodies were simultaneously incubated (cFos/CaMKII, cFos/GAD67), then the secondary antibodies, cy3-conjugated donkey anti-rabbit IgG and cy2-conjugated donkey anti-mouse, were used to detect the primary antibodies. The total number of c-Fos-positive nuclei were calculated and tested with an unpaired t test.

DREADD: To detect placement of the DREADD, fresh perfused (4% PFA) mouse brain was collected, cryoprotected in 30% sucrose in 0.1M PBS, and sectioned on a sliding microtome (50 µm). Every 6^th^ free-floating coronal section of hippocampus was quenched free of endogenous peroxidases in 0.3% H_2_O_2_, and blocked in 3% normal goat serum, prior to overnight incubation of rabbit anti-mCherry polyclonal antibody (1:500; Thermo Scientific). Primary antibody was detected by sequential incubation with biotinylated goat anti-rabbit IgG (1:1000, Jackson ImmunoResearch) and avidin-biotin complex (Vector Laboratories). Diaminobenzidine chromogen was used to detect the immunoperoxidase signal. Mice which displayed mCherry signal in the dorsal or ventral CA3 were included in analysis.

**Molecular Analyses:**

Western blots were carried out on micro-dissected hippocampal subfield tissue to analyze subfield-specific molecular markers in mice (n=7/group) as previously reported (50). β-Tubulin immunoreactivity was used as a loading control. Antibodies were obtained commercially: GluN2A and GluN2B (Millipore, CA), GluN1 (R&D systems), β-Tubulin (Thermoscientific, IL). Peroxidase-conjugated was the secondary antibody (1:10, 000) which enhanced chemiluminescence (Amersham NJ). Film-based images of immunoreactive bands were captured using Color Video Camera 3CCD Exwave HAD (SONY) and band intensities were analyzed by densitometry using Scion image software (version 1.62c). Immunoblotting data were analyzed using unpaired t-test.

**Discussion**

The hyperactive CA3-mediated associative memory function observed in KO mice is consistent with the role of CA3 in this memory process. Conversely, literature shows that CA3-lesioned rats have reduced contextual and cued fear conditioning (51;52). Moreover, the CA3-specific GluN1 KO mouse shows reduced freezing in contextual fear conditioning (53) and impaired rapid associative memory in a modified passive avoidance test (54). Mouse studies indicating that modulating MF pathway transmission compromises contextual fear memory (55-57), implicate abnormal fear memory with MF pathway pathology. Acknowledging the evidence that DG is a principal region in pattern separation function (58-60), we interpret the lack of memory accuracy seen in MWM as secondary to GluN1 depletion in DG in the KO mice, and consistent with impaired pattern separation in the KO during a modified FC paradigm (35). It is interesting that social cognition was also affected with CA3 hyperactivity in the DREADD preparation, suggesting functional relevance of the forward-driving circuit from CA3.
